# Carbohydrate Oral Rinsing, Cycling Performance and Individual Complex Carbohydrate Taste Sensitivity

**DOI:** 10.3390/nu16030459

**Published:** 2024-02-05

**Authors:** Claudia Hartley, Amelia Carr, Spencer S. H. Roberts, Wender L. P. Bredie, Russell S. J. Keast

**Affiliations:** 1 CASS Food Research Centre, Deakin University, Burwood Highway, Burwood, VIC 3125, Australia; c.hartley@deakin.edu.au; 2 Department of Food Science, University of Copenhagen, Rolighedsvej 26, 1958 Frederiksberg, Denmark; wb@food.ku.dk; 3 Centre for Sport Research, Deakin University, Burwood Highway, Burwood, VIC 3125, Australia; amelia.carr@deakin.edu.au (A.C.); s.roberts@deakin.edu.au (S.S.H.R.)

**Keywords:** carbohydrates, exercise performance, cycling, taste, taste sensitivity, oral rinsing

## Abstract

The aim of this pilot study was to determine the effect of individual complex carbohydrate taste sensitivity on cycling performance with complex carbohydrate oral rinsing. Ten male participants completed five cycling time trials in a fasted state with a seven-day washout period between each trial. Participants completed a fixed amount of work (738.45 ± 150.74 kJ) as fast as possible on a cycle ergometer while rinsing with an oral rinse for 10 s every 12.5% of the trial. An oral rinse (maltodextrin, oligofructose, glucose, sucralose or water control) was given per visit in a randomised, crossover, blinded design. Afterwards, participants had their taste assessed with three stimuli, complex carbohydrate (maltodextrin), sweet (glucose) and sour (citric acid), using taste assessment protocol to determine individual taste sensitivity status. Participants were subsequently grouped according to their complex carbohydrate taste sensitivity and complex carbohydrate taste intensity. There were no significant effects of the oral rinses on cycling performance time (*p* = 0.173). Participants who did not have improvements in exercise performance with the maltodextrin rinse experienced a stronger taste intensity with complex carbohydrate stimuli at baseline (*p* = 0.047) and overall (*p* = 0.047) than those who did have improvements in performance. Overall, a carbohydrate oral rinse was ineffective in significantly improving cycling performance in comparison with a water control. However, when participants were grouped according to complex carbohydrate taste intensity, differences in exercise performance suggest that individual sensitivity status to complex carbohydrates could impact the efficacy of a carbohydrate-based oral rinse.

## 1. Introduction

Carbohydrates are an essential source of fuel for exercise [1]. The impact of carbohydrate consumption on exercise performance was first investigated in 1920 when researchers discovered exercise was less fatiguing when fed a high-carbohydrate versus a high-fat diet [1]. Since then, ingestion of carbohydrates prior to or during endurance exercise has become a key dietary recommendation for endurance performance [2,3,4,5]. For optimal performance, the current recommendations suggest that during endurance exercise, 30–60 g of carbohydrates be ingested per hour, and for sustained high-intensity exercise, small amounts of carbohydrates be ingested (including oral rinsing) [6,7]. However, a disadvantage of carbohydrate ingestion during exercise is the possibility of gastrointestinal (GI) discomfort [3,8,9,10,11]. Specifically, among a group of cyclists, 67% experienced upper gastrointestinal symptoms (nausea, belching, chest pain, heartburn and vomiting) while 64% experienced lower gastrointestinal symptoms (GI cramps, bloating, diarrhoea and side aches) [12]. These symptoms can negatively affect exercise performance [7,12,13,14] and could hinder athletes from finishing or winning a race [12].

An alternative method of carbohydrate delivery during exercise is the use of a carbohydrate-based oral rinse. The research illustrates that carbohydrate oral rinsing can facilitate improvements in exercise performance. In previous research, a carbohydrate oral rinse led to improvements in both power output and performance time in cycling [15,16,17], running [18,19] and resistance exercise [20,21]. This is supported by a recent meta-analysis conducted by our research group that provides evidence that a maltodextrin-based oral rinse can improve overall exercise performance [22]. The meta-analysis demonstrated that for moderate to high-intensity exercise with a time span ranging from 30 to 75 min [3,5], the optimal protocol is to use a carbohydrate-based rinse for 10 s with a concentration between 6 and 6.5% [22]. However, little is known about the importance of individual taste sensitivity to carbohydrates or the importance of carbohydrate type and composition.

In recent years, sensory research has investigated the taste perception of non-sweet carbohydrates (complex carbohydrates). Although it is well established that sugars are detectable in the oral cavity (sweet taste) through the receptor T1R2/T1R3 [23], complex carbohydrates have been thought to be invisible to the human palate [24,25]. Taste perception can facilitate the decision of whether a food is safe for consumption. For example, sweet tastes can indicate readily available energy in food while strong bitter tastes can often indicate the presence of toxins in foods [26,27]. Taste is stimulated when taste receptor cells are activated by non-volatile, saliva-soluble chemicals [26,28]. This stimulation initiates a signal transduction to the parts of the brain, which enables taste perception [26,28]. In order to facilitate the absorption of carbohydrates in the human body, hydrolysis of the carbohydrate first occurs. Once carbohydrates enter the oral cavity, salivary α-amylase begins to hydrolyse the α-1,4 glycosidic linkages to produce monosaccharides and disaccharides [29]. The carbohydrates continue to break down throughout the digestive system where they are then absorbed into the bloodstream [30]. Recent human psychophysical research conducted by Low et al. has used taste assessment methodology (detection threshold and suprathreshold intensity perception) to determine that over a range of concentrations, complex carbohydrates (maltodextrin) can be perceived in the oral cavity [31,32]. By classifying participants as hypersensitive, normosensitive and hyposensitive to complex carbohydrates, research has illustrated that individual carbohydrate taste sensitivity can influence taste intensity, waist circumference, energy intake [33] and food consumption [31]. At present, no studies have examined the influence of taste sensitivity on the efficacy of carbohydrate oral rinsing for improving endurance performance. Investigating an athlete’s sensitivity status to complex carbohydrates may allow for a tailored carbohydrate supplementation strategy to allow for optimum exercise performance. For example, an athlete may be hypersensitive to complex carbohydrates and require a specific concentration or composition of carbohydrates in order to optimise exercise performance.

The type of carbohydrate used for rinsing is another factor that may influence the efficacy of a carbohydrate oral rinse for improving performance. Oral rinses used to investigate the effect on exercise performance are often composed of maltodextrin [15,16,34,35,36,37,38,39,40,41,42,43,44,45,46,47,48], glucose [16,17,36,38,40,49], sucrose [50] and sucralose [51]. While these oral rinses have been utilised in previous research, the effect of a sucralose oral rinse has not been compared with a maltodextrin-based oral rinse and the effect of an oligofructose oral rinse on exercise performance has not been investigated. It has been established that maltodextrin and oligofructose are strongly correlated with both detection threshold and suprathreshold intensity perception [32]. Those who were classified as more sensitive to maltodextrin were also more sensitive to oligofructose. This demonstrates possible similarities in terms of transduction pathways [32] and, hence, both complex carbohydrates may improve exercise performance to similar magnitudes. Furthermore, it is unknown if a link exists between an individual’s oral sensitivity to complex carbohydrates and exercise performance while using a carbohydrate oral rinse. Therefore, the aim of this study was to examine the efficacy of carbohydrate oral rinses for improving endurance performance and determine whether individual complex carbohydrate taste sensitivity influences the efficacy of carbohydrate rinses.

## 2. Materials and Methods

### 2.1. Participants

Sixteen male participants were recruited from across Melbourne, Victoria to take part in this study. A total of 10 participants completed the study (age (years): 29.0 ± 9.1, height (cm): 176.7 ± 7.9, body mass (kg): 78.2 ± 13.6, BMI (kg/m2): 24.9 ± 2.9, body composition (%): 15.4 ± 2.9). Additional participants were recruited but withdrew (*n* = 6) due to either scheduling issues or an unwillingness to complete the study. Each participant provided their written, informed consent to participate in the research and the protocol was approved by the Deakin University Human Research Ethics Committee (Project ID: 2019-446). This study was conducted in accordance with the Declaration of Helsinki.

The inclusion criteria for this study comprised: (1) male participants aged 18 years old to 50 years who participate in physical activity and (2) who had been fully vaccinated with a COVID-19 vaccine. At a minimum, participants were required to meet the minimum performance level (PL) of 1 [52]. This PL outlines minimum values for absolute peak power output (<280 W) and relative peak power output (<4.0 W/kg) [52].

Potential participants were excluded from this study if they: (1) were smokers; (2) had known food allergies; (3) had known impaired taste or smell function; (4) had a recent musculoskeletal injury or (5) had a heart or respiratory condition that would impact their exercise performance or prevent them from successfully completing the study.

### 2.2. Experimental Design

**Figure 1:**
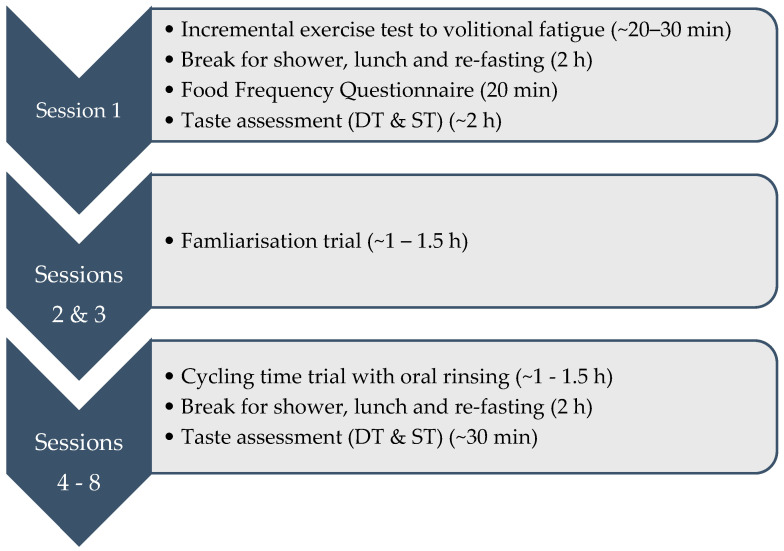
Overall diagram of sessions. Participants completed 8 sessions with a minimum 7-day washout period between each session.

Participants completed a total of eight testing sessions (see Figure 1 for an overview). Each session was conducted at a similar time in the day (±1 h) and separated by a washout period of a minimum of seven days [15]. Participants first completed a screening session, followed by two familiarisation sessions and then completed five experimental sessions conducted in a randomised, counterbalanced, crossover, blinded study design. All sessions were completed on an electrically braked cycle ergometer (Lode Excalibur, Groningen, The Netherlands) in a laboratory environment maintained at 22 ± 1 °C.

### 2.3. Session 1

In the first session, participants’ anthropometric data was collected. Body mass was measured to the nearest 0.1 kg using a segmental body composition analyser (TBF-300A) (Tanita Corporation, Tokyo, Japan). Height was measured to the nearest 0.1 cm using a portable stadiometer (Seca 213) (Seca, Hamburg, Germany). Body composition was measured using a segmental body composition analyser (TBF-300A) (Tanita Corporation, Tokyo, Japan). In a fasted state, participants then completed an exercise test to volitional fatigue to measure maximal workload (W_max_). W_max_ is defined as the total W of the final completed workload [15]. On a Lode cycle ergometer, participants commenced the test at 95 W which was increased by 35 W every three minutes until fatigue [15]. Heart rate (HR) was continuously recorded throughout the test. Each participant’s W_max_ value was used in the subsequent cycling time trials in sessions 2–8. Following a two-hour break, participants completed a Food Frequency Questionnaire (FFQ) (this version was adapted from the 1995 Australian National Nutrition Survey FFQ [53]) to quantify dietary intake. Also, participants had their taste function assessed.

#### 2.3.1. Sensory Stimuli

Prototypical stimuli for sweet (glucose, The Melbourne Food Depot, Melbourne, Australia) and sour (citric acid, Ward McKenzie Private Limited, Altona, Australia), were used to investigate the taste function (see Table 1). Oral carbohydrate taste sensitivity was measured for maltodextrin (DP 24, Star-Dri 5, Tate & Lyle Ingredients Americas, Decatur, IL, USA). For further details of stimuli, see Table 1 and Table 2. Prior to testing, solutions were prepared with filtered water and stored at room temperature (20 ± 1 °C) in glass beakers.

#### 2.3.2. Sensory Methods

Assessing participants’ taste function involved using reliable measures of taste perception routinely used in chemosensory research, which is as follows: (1) detection threshold (DT) and (2) suprathreshold intensity perception (ST). These measures were tested for two prototypical tastants (sweet and sour) and an additional tastant (maltodextrin for oral carbohydrate taste measurement). These measures were repeated for each tastant to ensure that accurate baseline taste measures were collected. Participants received each tastant in a randomised order. All taste assessment measures were conducted in individual, computerised, partitioned sensory booths in a laboratory environment. The sensory evaluation software Compusense Cloud version 23.0 (Compusense Inc., Guelph, ON, Canada) was used to collect the taste assessment measures for this study. All solutions were served at room temperature in a sample cup printed with a three-digit code for blinding purposes. Prior to each taste assessment, participants fasted for a minimum of 1 h.

Based on the International Standard Organisation (ISO) Method of Investigating Sensitivity of Taste [54] and previous research [32], DT was determined for each participant. For each tastant (Table 1), participants were provided with nine 15 mL samples presented with a three-digit randomised code and wore a nose clip during testing. These nine samples were arranged in ascending concentration (i.e., dilution 9 (the weakest concentration) to dilution 1 (the strongest concentration)). For each of the tastants, participants were asked if: (1) the sample tasted like water; (2) something other than water or (3) specific taste quality (sweet, salty, sour, bitter or umami). Between samples, participants rinsed their mouths with the filtered water provided [32]. DT was defined as the concentration at which the participants selected the ‘taste identified, but unknown taste quality’ response [54].

To determine ST for the two prototypical tastes (sweet and sour) and carbohydrate (maltodextrin), three concentrations (weak, medium and strong) and a blank (control) sample were used. Detailed in Table 2 are the tastant and stimuli concentrations used to determine ST. Participants received trays containing four concentrations of each tastant, the presentation order of each sample was randomised for each participant. All samples were presented with a three-digit randomised code and participants wore a nose clip during testing. Participants were instructed to place the 15 mL sample in their mouth and hold for it five seconds before expectorating. Using the Labelled Magnitude Scale (LMS), a psychophysical tool [55], participants rated the perceived intensity of each sample. The scale is a vertical line scale with accompanying descriptors ranging from ‘barely detectable’, ‘weak’, ‘moderate’, ‘strong’, ‘very strong’ and ‘strongest imaginable’. Between samples, participants rinsed their mouths with the filtered water provided [32]. In order to minimize any sensory or mental fatigue, participants were given multiple breaks and provided with water and crackers [56,57,58].

#### 2.3.3. Food Frequency Questionnaire

To quantify dietary intake, participants completed an adapted version of the 1995 Australian National Nutrition Survey FFQ [53] that has been used in previous research [58]. During the questionnaire, participants indicated on average how many times in the previous month they had consumed certain food and beverages, vitamin and mineral supplements. The questionnaire comprised 118 items divided into the following categories: bread and cereal foods; dairy foods; meat, fish and eggs; sweets, baked goods, and snacks; dressings; non-dairy beverages; vegetables and fruits. From this, participants were instructed to select an answer on a nine-point scale with response options ranging from ‘never or less than once per month’ to ‘6 or more times per day’. For the statistical analysis of the questionnaire, the response options for the consumption variables were collapsed [59]. For example, the rice category originally had nine response options and was recoded down to three response options.

### 2.4. Sessions 2–8

In sessions 2–8, participants completed cycling time trials. To determine the amount of work that was conducted in the cycling time trials, each participant’s W_max_ value from the incremental exercise test in the first session was used in a formula: Total Work (J) = 0.75 × W_max_ × 3600 [60]. Each participant’s alpha value was calculated from 75% of their W_max_ value (to determine resistance). After a five-minute warmup, participants were asked to complete the allocated amount of work (calculated from the above formula) (738.45 ± 150.74 kJ) as quickly as possible. Every 20% of the time trial, participants rated their perceived level of exertion (RPE) using the 6—to 20—point Borg Scale [61]. HR was continuously recorded throughout the test (Polar H10 Heart Rate Sensor, Polar Electro Oy, Kempele, Finland). Sessions two and three were familiarisation trials which allowed participants to develop a stable pacing strategy [62] and to limit any learning effects across the intervention [63]. In sessions four to eight, participants completed the cycling time trials with oral rinsing. During each trial, no encouragement was given to the participants and an opaque screen separated participants from each other. These methods were based on protocols used in previous research within the research area [15].

#### 2.4.1. Oral Rinse Protocol

For each time trial, a 25 mL oral rinse was given to participants for every 12.5% of the time trial completed, including the warmup [15]. Participants were required to rinse for 10 s before expectorating into a jug held by the researcher. Upon completion of the trial, the volume of expectorated solution was measured to ensure that ingestion had not occurred. The rinses were either maltodextrin (DP 24, Star-Dri 5, Tate & Lyle Ingredients Americas, Wilmington, DE, USA), oligofructose (Fibrulose F97, CoSucra-Groupe, Warcoing, Belgium), sucralose (The Melbourne Food Depot, Melbourne, Australia), glucose (The Melbourne Food Depot, Melbourne, Australia) or a water control. During the familiarisation sessions, (sessions 2 and 3), participants received a water (blank) rinse. The oral rinses were selected for the following reasons: maltodextrin and oligofructose were selected as complex carbohydrate rinses, sucralose was selected as a non-carbohydrate sweet rinse, glucose was selected as a carbohydrate sweet rinse and water was selected as a control. The concentrations of each oral rinse are presented in Table 3.

Participants received one type of rinse per visit and the order was determined by a randomised, balanced Latin Square sample set. Participants were blinded to the rinse composition using blinding methods over the course of the study. The oral rinse protocol was based on previous research that found that rinsing for 10 s at a concentration of between 6 and 6.5% was the optimum condition for performance increases in exercise [22].

#### 2.4.2. Dietary Procedures

For the 24 h prior to the testing sessions, participants were instructed to avoid high-intensity exercise to reduce pre-test fatigue [64,65] and to consume foods and fluids that were consistent with their usual intake to reduce the likelihood of confounding variables [66]. Participants arrived at the laboratory in a fasted state, with the ingestion of food or fluids prohibited (water permitted) for 10 h prior to each testing session.

#### 2.4.3. Sensory Methods

In sessions 4–8, participants had their taste further assessed using DT and ST methods. As described for the sensory methods in the first session, identical methods were used for sessions 4–8. These measures were repeated for the maltodextrin tastant to ensure accurate taste measures were collected. Table 1 and Table 2 illustrate the concentrations used for each tastant.

### 2.5. Statistical Analysis

Data were reported as mean and standard deviation (mean ± SD), unless otherwise stated. Statistical significance was accepted at *p* < 0.05 for all analyses. The analysis was carried out using Stata Statistical software version 16.0 (StataCorp LLC, College Station, TX, USA). The effect of the oral rinses on the outcomes (time to completion, power output, HR and RPE) was estimated using linear mixed models with the variables of the oral rinse and period (the order each oral rinse was received) as fixed effects and the participant as a random effect variable. Differences between body mass and body composition at the start of the trial and the end of the trial were analysed with a paired *t*-test. The rinse condition oligofructose was completed by *n* = 9 participants as during the trial, there was a malfunction with the Lode bike and those trial results were discarded. The other rinsing conditions were completed by *n* = 10 participants. Descriptive statistics were used to describe demographic information, complex carbohydrate DTs and STs. The DTs were calculated as the mean score of the repeated measures and a natural log transformation was applied. For ST, the mean of the three ratings (weak, moderate and strong) was calculated. DTs and STs for maltodextrin were treated as grouping variables with participants categorised as more sensitive to complex carbohydrates (1/2) or less sensitive to complex carbohydrates (2/2) to investigate differences between categorical (frequency of consumption of complex carbohydrate-based foods) variables. Fisher’s Exact test was used to detect differences in the frequency of consumption of complex carbohydrate-based foods and taste sensitivity status. A Kruskal–Wallis test was used to examine differences between DTs and STs for participants categorised as more sensitive to complex carbohydrates (1/2) or less sensitive to complex carbohydrates (2/2) at various time points across the intervention. Pearson’s product-moment correlations were conducted to analyse the relationship between oral complex carbohydrate taste sensitivity (DTs and STs) and mean performance time, mean power output and BMI. To further analyse differences between participants, they were either classified as ‘Complex Carbohydrate Responders’ (participants with improved exercise performance with the maltodextrin rinse) or ‘Complex Carbohydrate Non-Responders’ (participants who did not show improved exercise performance with the maltodextrin rinse).

## 3. Results

### 3.1. Performance Time and Power Output

**Figure 2:**
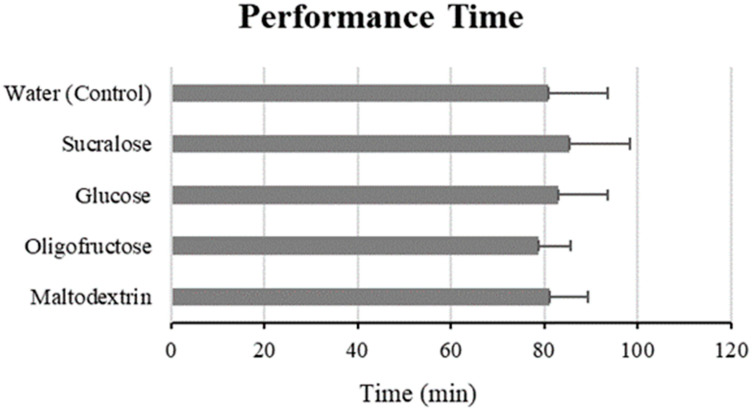
Mean performance time for each rinse condition. There was no significant effect of the oral rinses on performance time (*p* = 0.173).

Across the rinsing conditions, there was no significant effect of the oral rinses on performance time (*p* = 0.173) (Figure 2). The mean performance time of all the rinsing conditions was 79.09 ± 12.25 min. Table 4 compares the rinsing conditions, details the mean performance time and mean difference of each rinse to the water control condition. Across the rinsing conditions, there was no significant effect of the oral rinses on power output (*p* = 0.379). The mean power output across all of the rinsing conditions was 155.96 ± 36.03 W.

When examining the participants individually, only two participants had decreased performance time and increased power output for all rinsing conditions in comparison to the water control. One participant did not show decreases in performance time or increased power output for any rinsing conditions compared to the water control.

### 3.2. Heart Rate, Rating of Perceived Exertion and Body Mass

When comparing the oral rinses and the water placebo, there were no effects on mean HR (*p* = 0.146) or maximum HR (*p* = 0.330). Additionally, there was no effect of the oral rinses on RPE at the conclusion of the trial (*p* = 0.197). There were also no significant differences in body mass (78.2 ± 13.6 kg vs. 77.9 ± 13.1 kg, *p* = 0.432) or body composition (15.4 ± 2.9% vs. 16.0 ± 2.7%, *p* = 0.551) at the start of the study compared to the conclusion of the study.

### 3.3. Oral Detection Thresholds

Overall, there were no significant changes in the participant’s mean DT when comparing at baseline and across the intervention (sessions 4–8) (*p* > 0.05) (Table 5). For maltodextrin, there was no change in the participant’s mean DT values from baseline and across the intervention. However, when visually comparing the means from baseline and session 8, the participant’s mean DT did decrease, indicating that across the intervention, participants may have become more sensitive to complex carbohydrate stimuli. Conversely, for the sweet taste quality, there was a visual increase in the participants’ mean DT across the intervention, which suggests that participants may have become less sensitive to sweet stimuli by the end of the trial. In Table 5, mean (±SD) log DT values are presented for complex carbohydrate (maltodextrin), sweet and sour taste qualities. For complex carbohydrate at baseline, there was large individual variation in DT between participants ranging from −2.3 to 2.0 % (log) *w*/*v* (Table 5).

### 3.4. Oral Suprathreshold Intensities

Overall, there were no significant changes in the participants’ mean ST values when compared at baseline and during the intervention (sessions 4–8) (*p* > 0.05) (Table 6). When visually comparing the data, for the complex carbohydrate taste quality maltodextrin, there was an increase in the participant’s mean ST values from baseline across the intervention. Similarly to the DT results, this further suggests that from baseline across the intervention, participants experienced a stronger intensity for the complex carbohydrate tastant. For the taste qualities sweet and sour, there was a visual decrease in mean ST across the trial which suggests that participants experienced a lower intensity to those taste qualities by the final session of the intervention.

### 3.5. Taste Sensitivity and Time Trial Performance

To examine the effects of the oral rinse and performance time and power output on carbohydrate taste sensitivity, participants were divided into two groups. Group 1 (*n* = 5) was labelled ‘Complex Carbohydrate Responders’, participants who had decreased time to completion and increased power output in the cycling time trials with the maltodextrin rinse in comparison to the water control rinse. Those who had an increased time to completion and decreased power output were allocated to Group 2 (*n* = 5) and labelled ‘Complex Carbohydrate Non-Responders’. This method of classification is based on similar research [67,68] This information is detailed in Table 7 and Table 8. There were no significant differences between the ‘Complex Carbohydrate Responders’ and ‘Complex Carbohydrate Non-Responders’ for mean DT or mean ST (*p* > 0.05). Due to the high variance between participants, a Kruskal–Wallis test was also used. This showed that there were significant differences in median ST at baseline between the two groups (*p* = 0.047) and significant differences in overall (baseline and intervention) median ST (*p* = 0.047) between the two groups. This suggests that at baseline, and overall, ‘Complex Carbohydrate Non-Responders’ experienced higher intensity and were more sensitive to carbohydrate stimuli than ‘Complex Carbohydrate Responders’.

### 3.6. Dietary Data

There were no significant differences between the frequency of consumption of complex carbohydrates and Complex Carbohydrate Responder status (*p* > 0.05). When examining oral complex carbohydrate taste sensitivity and the frequency of consumption of carbohydrates, there were no significant differences with participants with a low overall DT compared with participants with a high overall DT (*p* > 0.05). However, for overall ST for complex carbohydrate, there were significant differences in consumption of white bread, toast or rolls (*p* = 0.008), noodles (*p* = 0.048), cakes, sweet muffins, scones or pikelets (*p* = 0.048), sweet pies or sweet pastries (*p* = 0.008) between participants with a low overall ST and a high overall ST. Participants with a high overall ST had a more frequent consumption of those complex carbohydrate-based food items than those with a low overall ST. Hence, this suggests that those who experience a higher intensity to complex carbohydrates consume certain complex carbohydrate-based foods more frequently than those who experience a lower intensity.

### 3.7. Correlations

No significant correlations were identified for complex carbohydrate DT or ST and cycling performance time with the carbohydrate oral rinse (r = 0.42 and r = −0.33, *p* > 0.05). No significant correlations were identified for carbohydrate DT or ST and cycling power output with the carbohydrate oral rinse (r = 0.15 and r = −0.11, *p* > 0.05). No significant correlations were found between DT or ST and BMI (r = 0.29 and r = 0.25, *p* > 0.05).

## 4. Discussion

The purpose of this study was to investigate the effect of various oral rinses on exercise performance and to investigate if a link existed between individual complex carbohydrate oral taste sensitivity and exercise performance. Specifically, this study investigated the effect of a maltodextrin, oligofructose, sucralose and glucose oral rinse compared to a water control on cycling performance (time to completion and power output) during a cycling time trial. Furthermore, this is the first study to investigate the effects of complex carbohydrate individual taste sensitivity on this relationship between oral rinsing and cycling performance.

In a fasted state, there were no significant differences in performance time or power output with the oral rinse conditions compared to the water control (*p* > 0.05). Additionally, there were no significant differences in HR or RPE between trials (*p* > 0.05). These findings indicate that for these participants, using an oral rinse during a cycling time trial did not improve exercise performance, physiological or perceptual responses. These findings follow the previous literature where, during a cycling time trial, no improvements in performance time [39,69] or power output [39,47,69,70] were found with a maltodextrin-based oral rinse. However, this is in contrast to the published literature which has found that a carbohydrate-based oral rinse improves cycling performance time [15,16,35,45] and power output [15,16,71].

In the current study, 10 participants completed five cycling time trials and were provided with an oral rinse to rinse for 10 s every 12.5% of the trial completed. All trials were completed at approximately the same time of day and participants had fasted for 10 h prior to the trial commencing. Trials were completed with a minimum of seven days in between sessions as a washout period. The protocol used in this study was based on previous research [15] and findings from a meta-analysis that demonstrated that rinsing a maltodextrin-based oral rinse for 10 s at a concentration of 6–6.4% were the optimal conditions for improvements in exercise performance [22]. However, despite employing the optimal protocol according to the existing research, no significant decreases in cycling performance time or increases in power output were evident with a complex carbohydrate-based oral rinse in comparison with the control.

**Figure 3:**
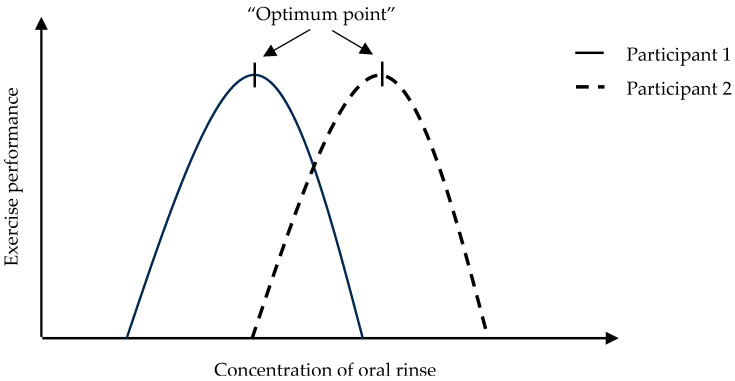
Hypothesised inverted U curve relationship of exercise performance and concentration of the complex carbohydrate oral rinse and corresponding “optimum point”. This “optimum point” may be different for each individual according to their complex carbohydrate taste sensitivity status.

An explanation for the variability between participants’ exercise performance outcomes could be the variation in complex carbohydrate taste perception or the corresponding oral receptors. From previous research, it can be argued that activation of the receptors involved in complex carbohydrate perception increases the consumption of complex carbohydrates [31,33]. From this, it could therefore be hypothesised that the activation of these receptors may inform the brain of the presence of carbohydrates and thereby increase exercise performance. When participants were grouped according to their Complex Carbohydrate Responder status, the ‘Complex Carbohydrate Non-Responders’ (defined as participants with an increased performance time and decreased power output with the maltodextrin oral rinse vs. water control) experienced a higher (stronger) taste intensity to complex carbohydrate stimuli (maltodextrin) at both baseline and overall than the ‘Complex Carbohydrate Responders’. This illustrates that perhaps for those who experience a higher taste intensity to complex carbohydrate stimuli, the standard 6.4% maltodextrin-based solution may not yield an improvement in exercise performance and they require a more tailored carbohydrate delivery system during exercise. Perhaps a different type of maltodextrin (e.g., with a longer chain length and increased level of sweetness) could be beneficial for these participants. Furthermore, similarly to a bliss point (a point at which an optimum concentration reaches maximal sensory liking [57]), people may have a varying “optimum point” to achieve optimum exercise performance results with a complex carbohydrate oral rinse. This relationship between performance and concentration of the oral rinse may follow an inverted U-shape relationship (Figure 3). As the concentration of the oral rinse increases, exercise performance also gradually increases until it reaches an “optimum point”. After this point, as the concentration continues to increase, exercise performance is impaired. However, this “optimum point” may be different for each individual depending on their complex carbohydrate taste sensitivity status.

One explanation for the differences observed between ‘Complex Carbohydrate Responders’ and ‘Complex Carbohydrate Non-Responders’ could be due to salivary α-amylase. As salivary α-amylase breaks down the maltodextrin oral rinse in the oral cavity, the α-1,4 glycosidic linkages are hydrolysed, resulting in the production of maltose (DP 2), maltotriose (DP 3) and larger oligosaccharides [29]. Salivary α-amylase levels can vary among individuals and can change with exercise [72,73,74], diet [75] and environmental factors (i.e., stress levels) [72,76]. Perhaps those who experience a higher taste intensity of complex carbohydrates have less active salivary α-amylase and the maltodextrin is broken down at a slower rate. This could, therefore, cause a slower or delayed response to the oral rinse during exercise and, therefore, not improve exercise performance. Future studies should investigate this hypothesis.

Data from this study showing no overall effect of carbohydrate rinsing on endurance exercise performance may also reflect the number of participants with hypersensitivity to complex carbohydrates. While the exact mechanism that is involved in improvements in exercise performance with carbohydrate oral rinsing is unknown, it is speculated that improved exercise performance is caused by the activation of higher brain regions. These higher brain regions are thought to link the corresponding cognitive, behavioural and emotional responses and the gustatory pathways [77,78]. Additionally, research has shown that these specific regions can be activated by oral exposure to carbohydrates but not by non-nutritive sweeteners [16,79,80], and this may support the explanation of the improvements in exercise performance in the literature. An alternative theory is that changes in exercise performance may be influenced by a mechanism that maintains homeostasis during exercise, the ‘Central Governor’ [81]. This mechanism is hypothesised to use afferent signals from peripheral physiological receptors and systems that detect changes in the external and internal environment to change power output [81]. Therefore, during exercise, it could be interpreted that the carbohydrate oral rinse generates positive central responses which could possibly counteract the negative physical, metabolic and thermal afferent signals [82]. However, if the participant is hypersensitive to complex carbohydrate stimuli, perhaps those positive central responses are not being generated at the same level as someone who is hyposensitive to complex carbohydrate stimuli, and this may contribute to the lack of improvements in exercise performance with a carbohydrate oral rinse.

In previous research where the maltodextrin rinse was effective in reducing cycling performance time [15,16,35,45] or increasing power output [15,16,71], perhaps the majority of participants experienced a lower intensity to complex carbohydrate stimuli and this is why improvements in performance time were seen. In the future, to ensure optimal exercise performance for all participants, it could be beneficial to screen participants’ sensitivity level to complex carbohydrates prior to exercise and additionally to determine their “optimum point” (Figure 3).

Furthermore, the type of maltodextrin and its chain length and degree of polymerisation could potentially affect the efficacy of a maltodextrin-based oral rinse. Subsequently, this is the first research article that has discussed the role of maltodextrin in an oral rinse during exercise. In a previous meta-analysis of 35 articles, 22 articles mentioned the brand or manufacturer details, while only 3 articles detailed the composition of the maltodextrin [22]. Maltodextrins with varying chain lengths, degree of polymerisation (DP) and dextrose equivalent (DE) have different taste properties. Shorter chain maltodextrins have a lower DP and higher DE and, therefore, have an increased sweetness [83], while in comparison, longer chain maltodextrins have a higher DP and lower DE, a more savoury taste [84] and a more viscous mouthfeel [85]. From this lack of information across the literature, it is not possible to disregard that the type of maltodextrin used in the oral rinse could be a confounding factor affecting the efficacy of the oral rinse itself. From this, it is important to investigate which type of maltodextrin and its corresponding DE and DP levels consistently provide improvements in exercise performance across all participants and to subsequently use that as a standard in an oral rinse.

### Limitations

Although there were novel aspects of this study, limitations exist. For this study, there were difficulties in recruiting participants and retaining participants. This resulted in the study having a smaller sample size of *n* = 10. However, previous studies investigating oral rinsing and cycling performance have used samples of similar size [15,16,35,45,48,71]. Also, some of the participants had a lower training status and limited cycling ability which could have resulted in some variability. Nonetheless, this study was able to highlight with this sample size and group of participants that individual complex carbohydrate taste sensitivity could impact the efficacy of carbohydrate oral rinsing.

## 5. Conclusions

In conclusion, the present study did not find an oral rinse to be effective in significantly improving exercise performance in a cycling time trial in comparison with a water control. When analysing participants as groups, the ‘Complex Carbohydrate Non-Responders’ experienced a higher taste intensity to complex carbohydrate stimuli. This highlights that individual sensitivity status to complex carbohydrates could be a predictor for the efficacy of a carbohydrate-based oral rinse. Further work should be conducted to establish this relationship more firmly.

## Figures and Tables

**Table 1:** Tastant stimuli concentrations used for the determination of detection thresholds for sweet (glucose), sour (citric acid) and complex carbohydrate (maltodextrin) stimuli.

Taste Quality	Stimulus	Concentration (% *w*/*v*)								
		1	2	3	4	5	6	7	8	9
Sweet	Glucose	0.05	0.09	0.1	0.2	0.4	0.6	1.1	1.8	2.9
Sour	Citric Acid	0.013	0.016	0.020	0.025	0.031	0.038	0.048	0.06	0.10
Carbohydrate	Maltodextrin	0.1	0.2	0.3	0.6	1.1	1.9	3.6	6.3	11.2

This concentration series for sweet, sour and carbohydrate taste qualities was used in previous research [32] and based on ISO methods [54]. The concentration series ranges from 1 to 9 (1: weakest concentration to 9: strongest concentration).

**Table 2:** Tastant stimuli concentrations used for the determination of suprathreshold taste intensity for sweet (glucose), sour (citric acid) and complex carbohydrate (maltodextrin) stimuli.

Taste Quality	Stimulus	Concentration (% *w*/*v*)		
		Weak	Medium	Strong
Sweet	Glucose	5.3	10.6	21.2
Sour	Citric Acid	0.02	0.06	0.13
Carbohydrate	Maltodextrin	3.6	6.3	11.2

This concentration series was used in previous research [32].

**Table 3:** Oral rinse concentrations used during cycling time trials. Oral rinses were presented in a randomised, counterbalanced, crossover, blinded design.

Rinse	Concentration (% *w*/*v*)
Maltodextrin	6.4
Oligofructose	6.4
Glucose	6.4
Sucralose	0.0057

This concentration series is based on previous research [15,16,17,22,32].

**Table 4:** Comparison of oral rinsing conditions for mean performance time (min) and SD, mean difference to control and confidence intervals (CI).

Oral Rinse	Mean Time (min)	Mean Difference Compared to Control	Confidence Interval (CI)
Water (Control)	81.00 ± 12.48	-	-
Sucralose	85.29 ± 13.22	+3.61	−2.26–9.48
Glucose	82.90 ± 10.61	+0.98	−4.85–6.81
Oligofructose	78.68 ± 6.86	−3.99	−10.14–2.16
Maltodextrin	81.25 ± 8.09	+0.58	−5.27–6.42

**Table 5:** Log detection thresholds for complex carbohydrate (maltodextrin), sweet (glucose) and sour (citric acid) taste qualities (% *w*/*v*), including mean, standard deviation (SD) and range at baseline, final session (session 8) and across the intervention (sessions 4–8).

Taste Quality	Timepoint	(log) Mean ± SD	(log) Range
Maltodextrin	Baseline	−0.6 ± 1.6	−2.3–2.0
	Session 8	−1.0 ± 1.4	−2.3–1.2
	Intervention	−0.5 ± 1.2	−2.3–1.3
Sweet	Baseline	−1.9 ± 1.1	−3.0–0.2
	Session 8	−1.7 ± 1.6	−3.0–1.1
	Intervention	−1.4 ± 1.3	−3.0–0.3
Sour	Baseline	−4.3 ± 0.0	-
	Session 8	−4.3 ± 0.0	-
	Intervention	−4.3 ± 0.01	−4.34–−4.30

**Table 6:** Suprathreshold intensity ratings for complex carbohydrate (maltodextrin), sweet (glucose) and sour (citric acid) taste qualities (% *w*/*v*), including mean, standard deviation (SD) and range at baseline, final session (session 8) and overall across the intervention (sessions 4–8).

Taste Quality	Timepoint	Mean ± SD	Range
Maltodextrin	Baseline	6.9 ± 3.9	0.7–13.2
	Session 8	12.6 ± 11.5	0.7–31.3
	Intervention	11.9 ± 10.6	2.3–35.6
Sweet	Baseline	39.7 ± 15.4	20.2–69.8
	Session 8	29.5 ± 12.7	7.7–53.2
	Intervention	35.6 ± 9.5	22.9–56.9
Sour	Baseline	42.8 ± 18.1	15.1–77.0
	Session 8	43.6 ± 12.8	26.8–61.9
	Intervention	41.5 ± 13.6	24.8–64.5

**Table 7:** Log detection thresholds for complex carbohydrate (% *w*/*v*), including mean, standard deviation (SD) and range for ‘Complex Carbohydrate Responders’ and ‘Complex Carbohydrate Non-Responders’ at baseline, final session (session 8), across the intervention (sessions 4–8) and overall (sessions 1–8).

		(log) Mean ± SD	(log) Median ± SD	(log) Range
Complex Carbohydrate Responders	Baseline	−0.3 ± 1.4	−0.4	−2.3–1.7
	Session 8	−0.5 ± 1.4	−0.1	−2.1–1.3
	Intervention	−1.3 ± 1.4	−1.9	−2.3–1.2
	Overall	−0.1 ± 1.1	0.2	−1.3–1.1
Complex Carbohydrate Non-Responders	Baseline	−0.9 ± 1.8	−1.4	−2.3–2.0
	Session 8	−0.6 ± 1.1	−0.5	−2.3–0.7
	Intervention	−0.8 ± 1.5	−0.4	−2.3–1.0
	Overall	−0.6 ± 1.2	−0.6	−2.3–1.1

**Table 8:** Suprathreshold intensity ratings for complex carbohydrate (% *w*/*v*), including mean, standard deviation (SD) and range for ‘Complex Carbohydrate Responders’ and ‘Complex Carbohydrate Non-Responders’ at baseline, final session (session 8), across the intervention (sessions 4–8) and overall (sessions 1–8).

		Mean ± SD	Median	Range
Complex Carbohydrate Responders	Baseline	4.8 ± 2.9	6.7 *	0.7–6.8
	Session 8	7.0 ± 8.9	4.1	0.7–22.3
	Intervention	6.9 ± 5.4	6.0	2.3–16.1
	Overall	6.6 ± 4.5	6.2 ^#^	2.0–13.9
Complex Carbohydrate Non-Responders	Baseline	9.0 ± 3.8	8.585 *	3.8–13.2
	Session 8	18.1 ± 11.9	18.4	4.3–31.3
	Intervention	16.8 ± 12.7	9.7	6.6–35.6
	Overall	15.5 ± 10.3	9.3 ^#^	7.7–30.3

*, **^#^** indicates significant difference between marked values (*p* < 0.05).

## Data Availability

The datasets generated during and/or analysed during the current study are available from the corresponding author on reasonable request. The data are not publicly available due to privacy and ethical reasons.
